# A set of electroencephalographic (EEG) data recorded during amplitude-modulated transcranial alternating current stimulation (AM-tACS) targeting 10-Hz steady-state visually evoked potentials (SSVEP)

**DOI:** 10.1016/j.dib.2021.107011

**Published:** 2021-03-29

**Authors:** David Haslacher, Khaled Nasr, Stephen E. Robinson, Christoph Braun, Surjo R. Soekadar

**Affiliations:** a Clinical Neurotechnology Laboratory, Neuroscience Research Center (NWFZ), Department of Psychiatry and Psychotherapy, Charité-University Medicine Berlin, Berlin, Germany; b National Institute of Mental Health (NIMH), MEG Core Facility, Bethesda, United States; c MEG Center, University of Tübingen, Germany; d CIMeC, Center of Mind/Brain Sciences, University of Trento, Italy

**Keywords:** Stimulation artifact source separation, Brain oscillations, Single-trial traces, Transcranial alternating current stimulation (tACS), Stimulation artifact, Electroencephalography (EEG)

## Abstract

Transcranial alternating current stimulation (tACS) can affect perception, learning and cognition, but the underlying mechanisms are not well understood. A promising strategy to elucidate these mechanisms aims at applying tACS while electric or magnetic brain oscillations targeted by stimulation are recorded. However, reconstructing brain oscillations targeted by tACS remains a challenging problem due to stimulation artifacts. Besides lack of an established strategy to effectively supress such stimulation artifacts, there are also no resources available that allow for the development and testing of new and effective tACS artefact suppression algorithms, such as adaptive spatial filtering using beamforming or signal-space projection. Here, we provide a full dataset comprising encephalographic (EEG) recordings across six healthy human volunteers who underwent 10-Hz amplitude-modulated tACS (AM-tACS) during a 10-Hz steady-state visually evoked potential (SSVEP) paradigm. Moreover, data and scripts are provided related to the validation of a novel stimulation artefact suppression strategy, Stimulation Artifact Source Separation (SASS), removing EEG signal components that are maximally different in the presence versus absence of stimulation. Besides including EEG single-trial data and comparisons of 10-Hz brain oscillatory phase and amplitude recorded across three conditions (condition 1: no stimulation, condition 2: stimulation with SASS, condition 3: stimulation without SASS), also power spectra and topographies of SSVEP amplitudes across all three conditions are presented. Moreover, data is provided for assessing nonlinear modulations of the stimulation artifact in both time and frequency domains due to heartbeats. Finally, the dataset includes eigenvalue spectra and spatial patterns of signal components that were identified and removed by SASS for stimulation artefact suppression at the target frequency. Besides providing a valuable resource to assess properties of AM-tACS artifacts in the EEG, this dataset allows for testing different artifact rejection methods and offers in-depth insights into the workings of SASS.

## Specifications Table

**Table:** 

Subject	Neuroscience (General)
Specific subject area	Neuroimaging and non-invasive brain stimulation
Type of data	Raw Electroencephalographic Data Graphs Figures
How data were acquired	EEG data were acquired with a 64-channel NeurOne system (Bittium Corp., Oulu, Finland). Amplitude-modulated transcranial alternating current stimulation (AM-tACS) was applied to the scalp of healthy human volunteers using a DC-Stimulator PLUS (NeuroConn GmbH, Ilmenau, Germany). Visual stimuli were presented via an Oculus Go (Oculus VR Inc., California, USA).
Data format	Raw Analyzed
Parameters for data collection	EEG was recorded in DC mode with a dynamic range of +/−430 V, a resolution of 51 nV/bit, and a range of 24 bit. It was ensured that electrode impedances stayed below 10 kOhm. EEG was sampled at 500 Hz with an anti-aliasing filter at 125 Hz. AM-tACS was applied through rubber electrodes placed over position CPz and on the inion. AM-tACS was applied at 2 mA (peak-to-peak), using a carrier frequency of 220 Hz and an envelope frequency of 10 Hz. Visual stimuli consisted of sinusoidal gratings flickering at 10 Hz.
Description of data collection	Initially, 64-channel EEG was recorded during a calibration session consisting of the presentation of 200 trials of visual flicker in absence of AM-tACS. Then, a session of 200 trials of visual flicker was recorded while AM-tACS was applied over the parietooccipital cortex. Trials were 2 s in length and separated by an inter-trial interval randomly distributed between 0.5 and 1 s. Approximately 20 min of data was recorded for each participant. One participant was excluded due to a lack of discernible SSVEPs in absence of AM-tACS.
Data source location	Charité – Universitätsmedizin Berlin Berlin Germany
Data accessibility	Raw data, along with all figures and scripts, are accessible on Mendeley Data (http://dx.doi.org/10.17632/fzcmhhjs76.3). The analysis scripts are accessible on GitHub (http://dx.doi.org/10.5281/zenodo.4592224).
Related research article	Haslacher, D., Nasr, K., Robinson, S. E., Braun, C., & Soekadar, S. R. [1]. Stimulation artifact source separation (SASS) for assessing electric brain oscillations during transcranial alternating current stimulation (tACS). *Neuroimage, 228*, 117,571. https://doi.org/10.1016/j.neuroimage.2020.117571

## Value of the Data

• The presented dataset allows for testing and validating novel stimulation artefact suppression algorithms that allow for reliable reconstruction of brain oscillations during transcranial alternating current stimulation (tACS).
• The data provide in-depth insights to the workings of a novel artefact suppression algorithm, Stimulation Artifact Source Separation (SASS), that is real-time compatible, e.g., to adapt tACS parameters to ongoing brain oscillations.
• Availability of this dataset will improve benchmarking when comparing different stimulation artefact suppression strategies.
• The dataset allows researchers and practitioners who do not have the means to collect 64-channel electroencephalographic (EEG) data during the application of amplitude modulated tACS to engage in the development of new and effective artefact suppression algorithms and brain stimulation protocols.

## 1 Data Description

**Fig. 1:**
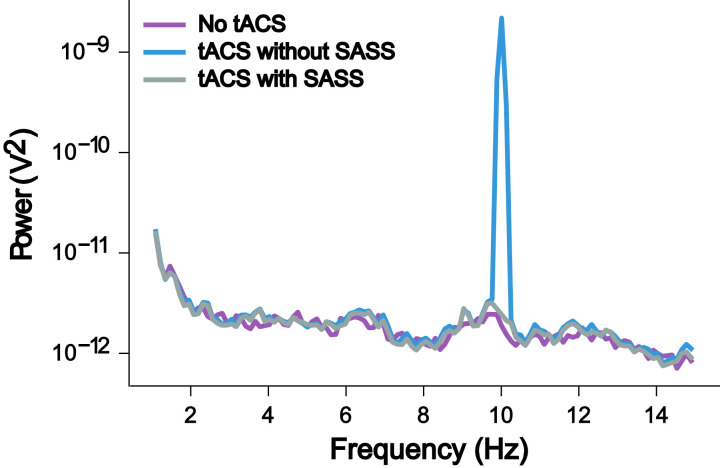
This plot for one representative participant depicts the Welch power spectral density of a virtual electroencephalographic (EEG) channel computed as the average of all available occipital electrodes. The equivalent plots (S1) for all participants are available on Mendeley Data at http://dx.doi.org/10.17632/fzcmhhjs76.3. These figures were published in an adapted form in [1].

**Fig. 2:**
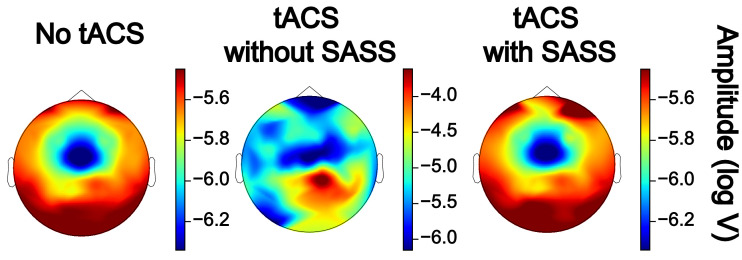
This plot for one representative participant depicts the mean amplitude of single steady-state visually evoked potential (SSVEP) trials as a topography over the entire sensor space. The equivalent plots (S2) for all participants are available on Mendeley Data at http://dx.doi.org/10.17632/fzcmhhjs76.3. These figures were published in an adapted form in [1].

**Fig. 3:**
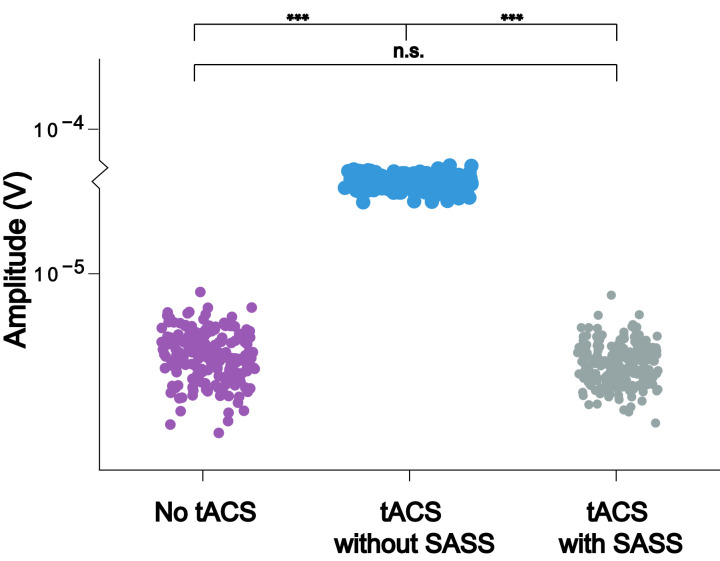
This plot for one representative participant depicts the amplitudes of single steady-state visually evoked potential (SSVEP) trials (*N* = 200) taken from a virtual electroencephalographic channel computed as the average of all occipital electrodes. To test for differences of single-trial amplitudes between the data in absence of AM-tACS and AM-tACS without SASS, one-sided t-tests for independent samples were employed. To test for differences between data in the presence of AM-tACS without SASS and AM-tACS with SASS, one-sided t-tests for dependent samples were employed. To test for differences between data in absence of AM-tACS and data in the presence of AM-tACS with SASS, two-sided t-tests for independent samples were employed. Significance levels are indicated as **** (*p* < 0.0001), *** (*p* < 0.001), ** (*p* < 0.01), or * (*p* < 0.05). A power analysis indicated that an effect of 0.281 (Cohen's d) could be detected, corresponding to a residual artifact (difference between single-trial amplitudes in absence of AM-tACS and in the presence of AM-tACS with SASS) of between 0.174 and 0.972 μV. The equivalent plots (S3) for all participants are available on Mendeley Data at http://dx.doi.org/10.17632/fzcmhhjs76.3. These figures were published in an adapted form in [1].

**Fig. 4:**
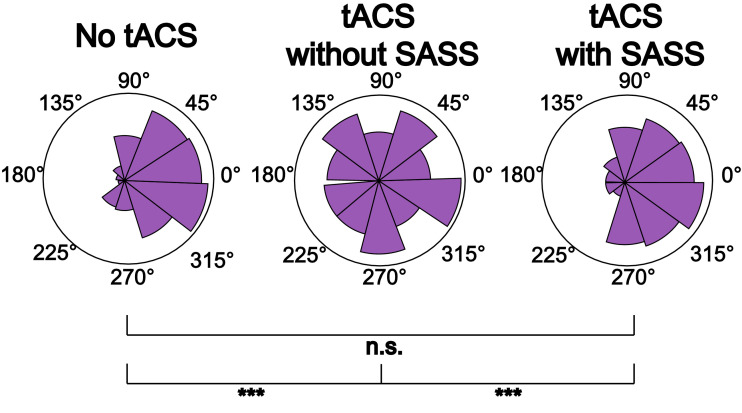
This plot depicts the phase (relative to flicker) of single steady-state visually evoked potential (SSVEP) trials (*N* = 200) taken from a virtual channel computed as the average of all occipital electrodes. To test for differences of single-trial phases between the data in absence of AM-tACS and AM-tACS without SASS or AM-tACS with SASS, Wallraff tests [11] for independent samples were employed, using Wilcoxon rank-sum test to compare angular distances. To test for differences between data in the presence of AM-tACS without SASS and AM-tACS with SASS, Wallraff tests for dependent samples were employed, using Mann-Whitney U tests to compare angular distances. Significance levels are indicated as **** (*p* < 0.0001), *** (*p* < 0.001), ** (*p* < 0.01), or * (*p* < 0.05). The equivalent plots (S4) for all participants are available on Mendeley Data at http://dx.doi.org/10.17632/fzcmhhjs76.3. These figures were published in an adapted form in [1].

**Fig. 5:**
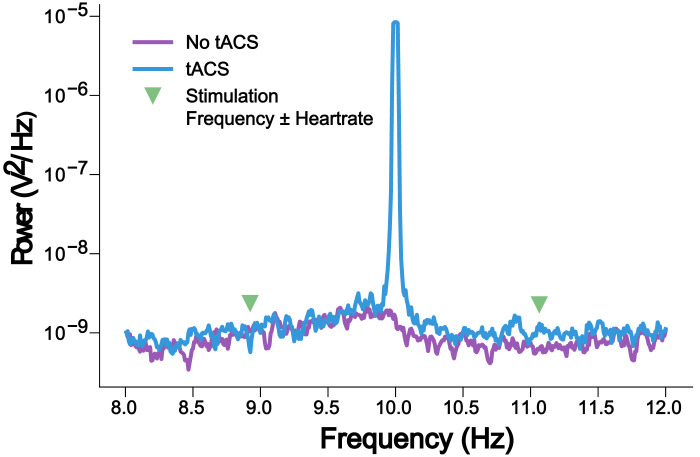
This plot for one representative participant depicts the high-resolution power spectrum of electrode O2. We have reproduced the time- and frequency-domain analyses of [12,13] around the amplitude-modulated transcranial alternating current stimulation (AM-tACS) target frequency. In contrast to conventional tACS, no such modulations in the power spectrum or in the time domain could be detected. The equivalent plots (S5) for all participants are available on Mendeley Data at http://dx.doi.org/10.17632/fzcmhhjs76.3. These figures were published in an adapted form in [1].

**Fig. 6:**
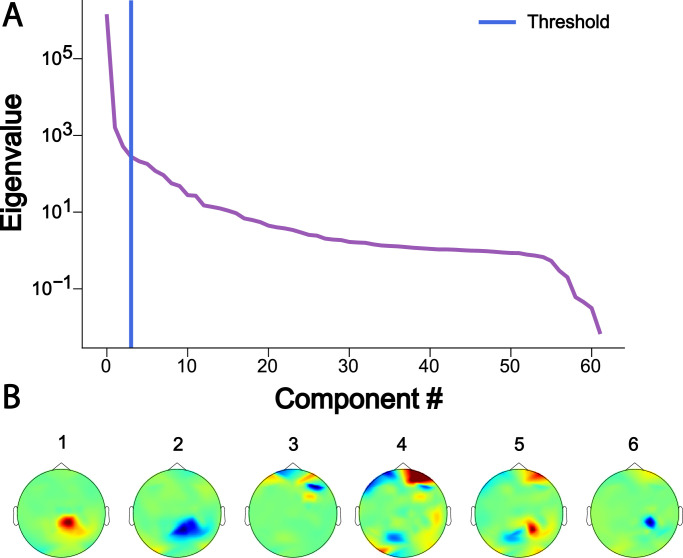
(A) This plot for one representative participant depicts the eigenvalue spectrum resulting from the joint diagonalization (generalized eigenvalue problem) of electroencephalographic sensor covariance matrices in absence of and during amplitude-modulated transcranial alternating current stimulation (AM-tACS), a procedure that forms the basis of Stimulation Artifact Source Separation (SASS). (B) The spatial patterns of the first six components resulting from the joint diagonalization procedure are depicted. The equivalent plots (S6) for all participants are available on Mendeley Data at http://dx.doi.org/10.17632/fzcmhhjs76.3.

**Fig. 7:**
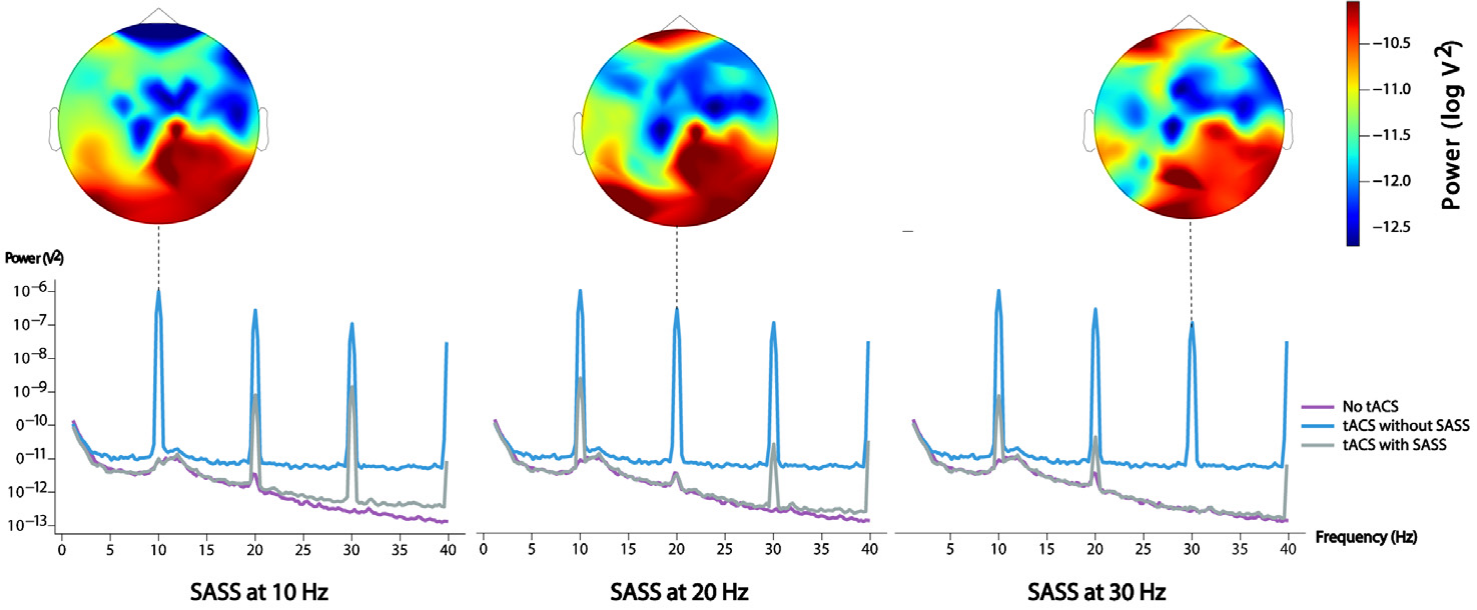
This plot depicts the spatial and spectral distribution of power when applying of Stimulation Artifact Source Separation (SASS) to different harmonics of the target frequency. It should be noted that the topography of stimulation artifacts is frequency-dependent (bottom). This is presumably caused by a spatially varying nonlinear transformations of the current by different capacitive effects at each electrode [13]. Therefore, SASS should be computed separately for each artifact frequency. This figure was published in an adapted form in [1].

**Fig. 8:**
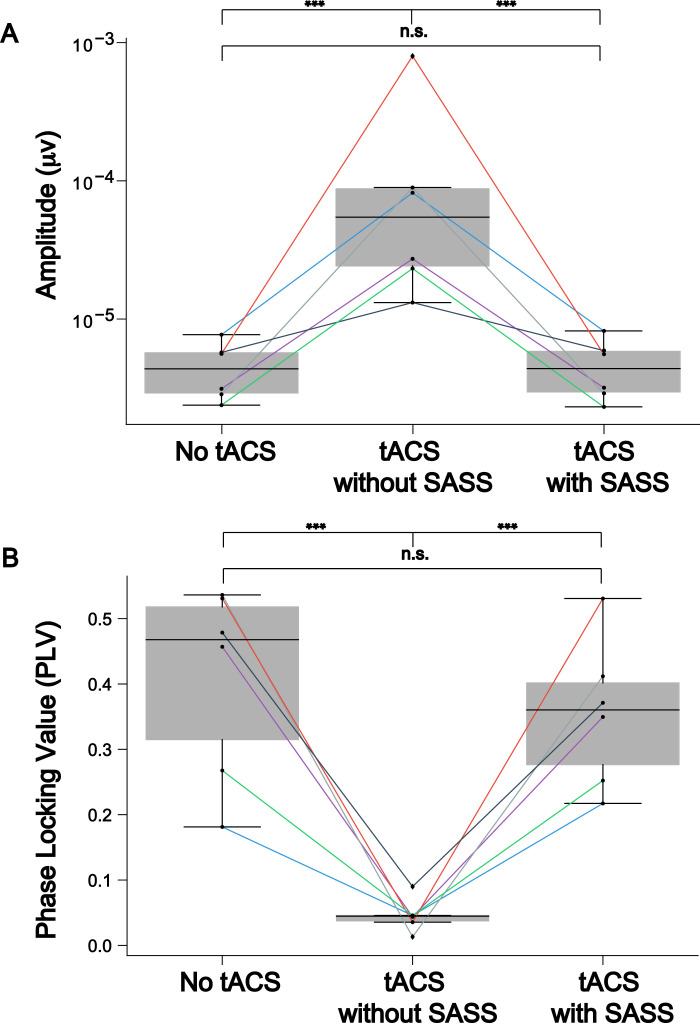
(a) This plot depicts group-level (*N* = 6) inter-trial phase locking value of steady-state visually evoked potentials (SSVEPs) when Stimulation Artifact Source Separation (SASS) is computed as usual on the full-length datasets. This figure was published in an adapted form in [1]. To test for differences of phase-locking values between the data in absence of AM-tACS and AM-tACS without SASS, or between AM-tACS without SASS and AM-tACS with SASS, a one-sided Wilcoxon rank-sum test was employed. To test for differences between data in absence of AM-tACS and data in the presence of AM-tACS with SASS, a two-sided Wilcoxon rank-sum test was employed. Significance levels are indicated as **** (*p* < 0.0001), *** (*p* < 0.001), ** (*p* < 0.01), or * (*p* < 0.05). (b) This plot depicts group-level (*N* = 6) mean amplitudes of steady-state visually evoked potentials (SSVEPs) when Stimulation Artifact Source Separation (SASS) is computed as usual on the full-length datasets. This figure was published in an adapted form in [1]. To test for differences of mean amplitudes between the data in absence of AM-tACS and AM-tACS without SASS, or between AM-tACS without SASS and AM-tACS with SASS, a one-sided Wilcoxon rank-sum test was employed. To test for differences between data in absence of AM-tACS and data in the presence of AM-tACS with SASS, a two-sided Wilcoxon rank-sum test was employed. Significance levels are indicated as **** (*p* < 0.0001), *** (*p* < 0.001), ** (*p* < 0.01), or * (*p* < 0.05).

**Fig. 9:**
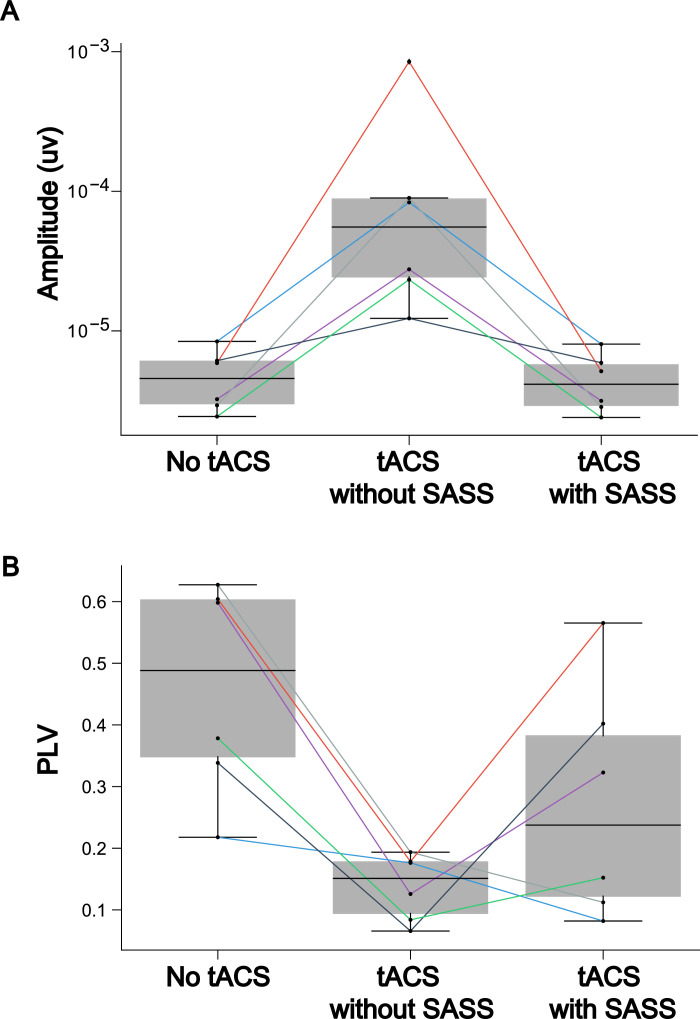
(a) This plot depicts group-level inter-trial phase locking value of steady-state visually evoked potentials (SSVEPs) when Stimulation Artifact Source Separation (SASS) is computed on the first half of the data with AM-tACS and applied to the second half. This figure was published in an adapted form in [1]. To test for differences of phase-locking values between the data in absence of AM-tACS and AM-tACS without SASS, or between AM-tACS without SASS and AM-tACS with SASS, a one-sided Wilcoxon rank-sum test was employed. To test for differences between data in absence of AM-tACS and data in the presence of AM-tACS with SASS, a two-sided Wilcoxon rank-sum test was employed. Significance levels are indicated as **** (*p* < 0.0001), *** (*p*< 0.001), ** (*p* < 0.01), or * (*p* < 0.05). (b) This plot depicts group-level mean amplitude of single-trial steady-state visually evoked potentials (SSVEPs) when Stimulation Artifact Source Separation (SASS) is computed on the first half of the data with AM-tACS and applied to the second half. This figure was published in an adapted form in [1]. To test for differences of mean amplitudes between the data in absence of AM-tACS and AM-tACS without SASS, or between AM-tACS without SASS and AM-tACS with SASS, a one-sided Wilcoxon rank-sum test was employed. To test for differences between data in absence of AM-tACS and data in the presence of AM-tACS with SASS, a two-sided Wilcoxon rank-sum test was employed. Significance levels are indicated as **** (*p* < 0.0001), *** (*p* < 0.001), ** (*p* < 0.01), or * (*p* < 0.05).

The EEG dataset described in the following was collected during the simultaneous application of 10-Hz AM-tACS and the presentation of 10-Hz visual flickers. This paradigm was used to validate SASS, a novel spatial filtering algorithm for rejection of AM-tACS artifacts in the EEG signal [1]. In this publication, it was shown that phase and amplitude of single-trial SSVEPs could be reconstructed in the presence of AM-tACS. While previous work focused on transcranial electric stimulation (TES) artifact rejection during magnetoencephalography (MEG) [2], [3], [4], [5], real-time compatible artifact rejection during EEG, e.g. in the context of brain-computer interface (BCI) applications [6], [7], [8] or closed-loop TES [9,10], has not been established yet. Figures depicting primary outcome measures (power spectra, topographies of 10-Hz power, single-trial 10-Hz phase and amplitude) associated with this result are included here for completeness (Fig. 1, Fig. 2, Fig. 3, Fig. 4, Fig. 5). Furthermore, we also provide additional figures illustrating the eigenvalue spectra and spatial patterns of components extracted from the signal by SASS (Fig. 6, Fig. 7, Fig. 8, Fig. 9). Finally, we provide the raw data and analysis scripts to reproduce all relevant outcome measures.

**p1/no_stim.eeg (.vhdr, .vmrk) – p6/no_stim.eeg (.vhdr, .vmrk)**

These files contain 64-channel EEG data featuring steady-state visually evoked potentials (SSVEPs) in absence of AM-tACS in the Brainvision format. Apart from channels in the standardized 10–20 system nomenclature, this dataset contains an ‘audio’ channel containing analogue pulses synchronized with the visual flicker.

**p1/open.eeg (.vhdr, .vmrk) – p6/open.eeg (.vhdr, .vmrk)**

These files contain 64-channel EEG data featuring steady-state visually evoked potentials (SSVEPs) during AM-tACS in the Brainvision format. Apart from channels in the standardized 10–20 system nomenclature, this dataset contains an ‘audio’ channel containing analogue pulses synchronized with the visual flicker.

**amplitudes_phases.py**

This script computes single-trial amplitudes (S3_1.pdf – S3_6.pdf) and phases (S4_1.pdf – S4_6.pdf) for each participant.

**filter_characteristics.py**

This script computes eigenvalue spectra (S6_1a.pdf – S6_6a.pdf) and topographies (S6_1b.pdf – S6_6b.pdf) of components found by Stimulation Artifact Source Separation (SASS) for each participant.

**group_amplitudes_phases.py**

This script computes the mean amplitude (S8a.pdf) and inter-trial phase-locking value (S8b.pdf) of single-trial steady-state visually evoked potentials (SSVEPs) for each participant when Stimulation Artifact Source Separation (SASS) is computed as usual on the full-length datasets.

**group_amplitudes_phases_validation.py**

This script computes the mean amplitude (S9a.pdf) and inter-trial phase-locking value (S9b.pdf) of single-trial steady-state visually evoked potentials (SSVEPs) for each participant when Stimulation Artifact Source Separation (SASS) is computed on the first half of data with AM-tACS and applied to the second half.

**heartbeat_modulation.py**

This script computes the high-resolution multitaper power spectral density (S5_1.pdf – S5_6.pdf) necessary to detect possible modulations of the stimulation artifact by heartbeats in the frequency domain.

**topoplot_amplitude.py**

This script computes the topographic plots of mean-single trial steady-state visually evoked potential (SSVEP) amplitude (S2_1.pdf – S2_6.pdf).

## 2 Experimental Design, Materials and Methods

### 2.1 Experimental design and electroencephalography (EEG) recording

EEG (64 channels, Bittium Corp., Oulu, Finland) was recorded from seven participants (22–28 years old) while they viewed white flickering gratings on a black background presented through a head-mounted display (Oculus VR Inc., California, USA). EEG was recorded in DC mode with a dynamic range of +/−430 V, a resolution of 51 nV/bit, and a range of 24 bit. It was ensured that electrode impedances stayed below 10 kOhm. Visual stimuli flickered at 10 Hz, and were presented for 2 s in each trial, with a random inter-trial interval of between 0.5 and 1 s. A trigger signal marking the flicker onset was fed into the EEG system to record stimulus timing. Two 10 min recording sessions were performed per participant, with a break of 5 min in between. In the first session, visual flickers were presented in absence of AM-tACS. In the second session, visual flickers were presented while AM-tACS targeting 10-Hz oscillations was applied. AM-tACS with a carrier frequency of 220 Hz, an envelope frequency of 10 Hz, and a peak-to-peak amplitude of 2 mA was applied through 4 × 5 cm rubber electrodes positioned over positions CPz and on the inion using a commercially available stimulator (NeuroConn GmbH, Ilmenau, Germany).

### 2.2 EEG data processing

The following describes the processing steps featured in the analysis scripts provided in the linked GitHub repository (see Specifications Table). The scripts, along with their output (i.e. the figures described in the Data Description section) are available along with the raw data on Mendeley Data (see Specifications Table). All analyses were implemented in MNE-Python [14]. Visual stimulus triggers were recorded as an audio channel, which was processed by z-scoring the channel. Subsequently, all occasions where the absolute z-score rose above 3 from below were obtained as events marking onset of the visual flicker. Individual flicker trials were segmented by finding flicker events without a preceding flicker event within 400 ms, as trials were separated by minimally 500 ms.

All EEG data was bandpass-filtered around 10 Hz using finite impulse response filters with a length of 1.65 s, which was used to compute SASS (see next section). The Hilbert transform was then applied to obtain sample-wise phase and amplitude of EEG signals at each electrode, which was then subsequently averaged within each trial to obtain single-trial phase and amplitude (amplitudes_phases.py). Phases obtained via the Hilbert transform were always transformed into the phase difference relative to the visual flicker before further analysis. Unless topographically plotted, these outcome measures were computed on a representative EEG channel computed as the average of all occipital sensors. For topographic representation of SSVEP amplitude (topoplot_amplitude.py), an average was taken across single trials for each participant and a log scale applied to allow for a visualization of artifact-cleaned and artifact-contaminated data on the same scale.

To obtain group-level measures of phase locking and amplitude of SSVEPs recovered by SASS (group_amplitudes_phases.py), we computed the phase-locking value [15], and mean amplitude across single trials. To validate the performance of SASS on a segment of data distinct from the one used to compute the covariance matrices (group_amplitudes_phases_validation.py), we computed the covariance matrices and SASS projection matrix on the first half of data of each participant (first 100 trials), and subsequently applied it to the second half of data (last 100 trials).

To assess modulation of the stimulation artifact by the heartbeat [12], we filtered the data from 5–15 Hz and applied the Hilbert transform to obtain the envelope (heartbeat_modulation.py). Then, 4 s segments centered on the ECG *R*-peak were demeaned and averaged. The significance of this average was tested at each timepoint by randomly placing the window centers 1000 times and computing the resulting permutation *p*-value, corrected for multiple comparisons. This procedure was performed independently for each channel.

To investigate the properties of the linear data decomposition described in the next section (joint eigenvalue problem of covariance matrices) forming the basis of SASS (filter_characteristics.py), we computed the eigenvalue spectra for each participant. The eigenvalue spectrum represented the ratio of power in the respective component in the condition in the presence versus absence of AM-tACS. We also plot the spatial patterns (rows of the matrix projecting from hidden space to data space) topographically.

### 2.3 Stimulation artifact source separation (SASS)

Covariance matrices B and A were computed without regularization separately from data in absence of and during AM-tACS, respectively. The projection matrix implementing stimulation artifact source separation(SASS) was computed fromthese two covariance matrices [1]. First, a joint diagonalization of the two sensor covariance matrices was performed:

$$W=\;\left(\begin{array}{c} w_{1^{T}}\\ \vdots \\ w_{n^{T}} \end{array}\right)\;\text{where}\;Aw_{i}=\lambda_{i}Bw_{i}\;\text{and}\;\lambda_{i}=\frac{w_{i}^{T}Aw_{i}}{w_{i}^{T}Bw_{i}}$$

Subsequently, the SASS projection matrix P implementing artifact rejection was computed:$P=W^{+}\mathrm{SW}$ where $S=\;\left(\begin{array}{ccc} 0 & & \\ & \ddots & \\ & & 1 \end{array}\right)$ and $W^{+}$ denotes the pseudoinverse of W

The number of rejected components (number of nulls) in the matrix S was chosen such that the mean squared difference of power across all sensors between cleaned data in the presence of AM-tACS and data in absence of AM-tACS was minimized.This projection matrix P was then applied to broadband EEG data to visualize power spectra, and to narrowband EEG data to compute single-trial phase and amplitude of 10 Hz SSVEPs.

## Declaration of Competing Interest

The authors declare that they have no known competing financial interests or personal relationships which have, or could be perceived to have, influenced the work reported in this article.

## Appendix Supplementary materials

Image, application 1

Image, application 2

Image, application 3

Image, application 4

Image, application 5

Image, application 6

## References

[1] Haslacher D., Nasr K., Robinson S.E., Braun C., Soekadar S.R. (2021). Stimulation artifact source separation (SASS) for assessing electric brain oscillations during transcranial alternating current stimulation (tACS). Neuroimage.

[2] Garcia-Cossio E., Witkowski M., Robinson S.E., Cohen L.G., Birbaumer N., Soekadar S.R. (2016). Simultaneous transcranial direct current stimulation (tDCS) and whole-head magnetoencephalography (MEG): assessing the impact of tDCS on slow cortical magnetic fields. Neuroimage.

[3] Soekadar S.R., Witkowski M., Cossio E.G., Birbaumer N., Robinson S.E., Cohen L.G. (2013). *In vivo* assessment of human brain oscillations during application of transcranial electric currents. Nat. Commun..

[4] Soekadar S.R., Witkowski M., Robinson S.E., Birbaumer N. (2013). Combining electric brain stimulation and source-based brain-machine interface (BMI) training in neurorehabilitation of chronic stroke. J. Neurol. Sci..

[5] Witkowski M., Garcia-Cossio E., Chander B.S., Braun C., Birbaumer N., Robinson S.E., Soekadar S.R. (2016). Mapping entrained brain oscillations during transcranial alternating current stimulation (tACS). Neuroimage.

[6] Nann M., Peekhaus N., Angerhöfer C., Soekadar S.R. (2020). Feasibility and safety of bilateral hybrid EEG/EOG brain/neural-maschine interaction. Front. Hum. Neurosci..

[7] Soekadar S.R., Witkowski M., Birbaumer N., Cohen L.G. (2015). Enhancing hebbian learning to control brain oscillatory activity. Cereb. Cortex.

[8] Soekadar S.R., Witkowski M., Gómez C., Opisso E., Medina J., Cortese M., Cempini M., Carrozza M.C., Cohen L.G., Birbaumer N., Vitiello N. (2016). Hybrid EEG/EOG-based brain/neural hand exoskeleton restores fully independent daily living activities after quadriplegia. Sci. Robot..

[9] Zrenner B., Zrenner C., Gordon P.C., Belardinelli P., McDermott E.J., Soekadar S.R., Fallgatter A.J., Ziemann U., Muller-Dahlhaus F. (2020). Brain oscillation-synchronized stimulation of the left dorsolateral prefrontal cortex in depression using real-time EEG-triggered TMS. Brain Stimul.

[10] Zrenner C., Belardinelli P., Muller-Dahlhaus F., Ziemann U. (2016). Closed-loop neuroscience and non-invasive brain stimulation: a tale of two loops. Front. Cell. Neurosci..

[11] Zar J.H. (1999). Biostatistical Analysis.

[12] Noury N., Hipp J.F., Siegel M. (2016). Physiological processes non-linearly affect electrophysiological recordings during transcranial electric stimulation. Neuroimage.

[13] Noury N., Siegel M. (2017). Phase properties of transcranial electrical stimulation artifacts in electrophysiological recordings. Neuroimage.

[14] Gramfort A., Luessi M., Larson E., Engemann D.A., Strohmeier D., Brodbeck C., Goj R., Jas M., Brooks T., Parkkonen L., Hämäläinen M. (2013). MEG and EEG data analysis with MNE-python. Front. Neurosci..

[15] Lachaux J.P., Rodriguez E., Martinerie J., Varela F.J. (1999). Measuring phase synchrony in brain signals. Hum. Brain Mapp..

